# A Circular Bioeconomy Approach to Using Post-Bioadsorbent Materials Intended for the Removal of Domestic Wastewater Contaminants as Potential Reinforcements

**DOI:** 10.3390/polym16131822

**Published:** 2024-06-27

**Authors:** Cristina E. Almeida-Naranjo, Alex Darío Aguilar, Vladimir Valle, Carlos Bastidas-Caldes, Alexis Debut, Britanny Sinchiguano

**Affiliations:** 1 Grupo de Biodiversidad Medio Ambiente y Salud (BIOMAS), Facultad de Ingeniería y Ciencias Aplicadas, Universidad de Las Américas, Redondel del Ciclista Antigua Vía a Nayón, Quito 170124, Ecuador; 2 Departamento de Ciencias de Alimentos y Biotecnología, Escuela Politécnica Nacional, Ladrón de Guevara E11-253, Quito 17-07-2759, Ecuador; alex.dario.aguilar@gmail.com; 3 Advanced Materials and Processes (MAP)—Technische Fakultät, Friedrich-Alexander-Universität Erlangen-Nürnberg, 91058 Erlangen, Germany; 4 Institute of Polymer Technology (LKT), Friedrich-Alexander-Universität Erlangen-Nürnberg, Am Weichselgarten 10, 91058 Erlangen-Tennenlohe, Germany; 5 One Health Research Group, Facultad de Ingeniería y Ciencias Aplicadas, Universidad de Las Américas, Redondel del Ciclista Antigua Vía a Nayón, Quito 170124, Ecuador; carlos.bastidas@udla.edu.ec; 6 Center of Nanoscience and Nanotechnology, Universidad de las Fuerzas Armadas ESPE, Sangolquí 17-15-231B, Ecuador; apdebut@espe.edu.ec; 7 Facultad de Ciencias, Ingeniería y Construcción, Universidad UTE, Rumipamba y Bourgeois, Quito 17-07-2759, Ecuador; britanny.sinchiguano@ute.edu.ec

**Keywords:** oil palm empty fruit bunch, mechanical properties, green composites, saturated bioadsorbents, agro-industrial residues valorization, circular bioeconomy

## Abstract

Agro-industrial residue valorization under the umbrella of the circular bioeconomy (CBE) has prompted the search for further forward-thinking alternatives that encourage the mitigation of the industry’s environmental footprint. From this perspective, second-life valorization (viz., thermoplastic composites) has been explored for agro-industrial waste (viz., oil palm empty fruit bunch fibers, OPEFBFs) that has already been used previously in other circular applications (viz., the removal of domestic wastewater contaminants). Particularly, this ongoing study evaluated the performance of raw residues (R-OPEFBFs) within three different size ranges (250–425, 425–600, 600–800 µm) both before and after their utilization in biofiltration processes (as post-adsorbents, P-OPEFBFs) to reinforce a polymer matrix of acrylic resin. The research examined the changes in R-OPEFBF composition and morphology caused by microorganisms in the biofilters and their impact on the mechanical properties of the composites. Smaller R-OPEFBFs (250–425 µm) demonstrated superior mechanical performance. Additionally, the composites with P-OPEFBFs displayed significant enhancements in their mechanical properties (3.9–40.3%) compared to those with R-OPEFBFs. The combination of the three fiber sizes improved the mechanical behavior of the composites, indicating the potential for both R-OPEFBFs and P-OPEFBFs as reinforcement materials in composite applications.

## 1. Introduction

**Figure 1:**
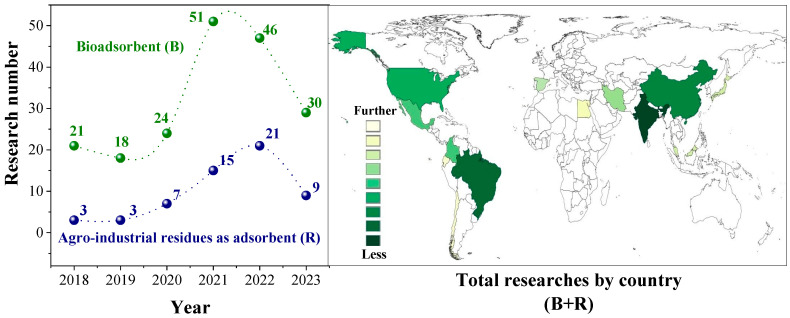
Research related to agro-industrial residues/bioadsorbents worldwide.

In the pursuit of sustainable development goals, the circular bioeconomy (CBE) (i.e., circular economy + biological resources) emerges as a pivotal concept, aiming to create sustainable systems by transforming liquid/solid waste into valuable resources. This approach not only addresses environmental concerns but also contributes to economic growth and social well-being. In wastewater treatment, the CBE not only optimizes water reuse and recovers nutrients but also generates renewable energy and develops valuable products [1]. On an annual basis, around 380 km^3^ of wastewater is produced worldwide, for which the treatment rate varies from ~8% to ~70% in developing and developed countries, respectively [2]. In this regard, it is noteworthy to point out that high treatment costs (e.g., ∼600 USD/(m^3^/d) by and large for a membrane bioreactor in China in 2018 [3]) drive the search for cost-effective alternatives. Indeed, within both developed and rural areas, filtration/biofiltration technologies are gaining traction, also aligning with the principles of the CBE and contributing to the objectives of sustainable development. Raw/modified agro-industrial residues (e.g., sugarcane bagasse, rice husks, oil palm empty fruit bunch fibers, etc.) have been assessed as potential filter beds [4]. For instance, a review of the last five years on Scopus using the keywords “biosorbents” and “agro-industrial residues” identified approximately 250 relevant research studies (Figure 1).

Even though the effective removal of contaminants (e.g., organic matter, nutrients, heavy metals, etc.) using filters/biofilters can be up to 100%, several key challenges, including the recovery and sustainable management of spent materials (post-adsorbents), have not yet been fully addressed. Indeed, less than 1% of Scopus publications explore applications for these spent materials. In this respect, typical methods for the use of these post-adsorbents are their regeneration through chemical/thermal/biological processes and microwave irradiation, among others. Nonetheless, beyond their overall endorsement, these processes still face a number of industrial-scale limitations due to their high energy requirements or their consumption of acids/bases/chelates/supercritical fluids [5]. In essence, as for regenerated adsorbents (i.e., less effective for contaminant removal by up to 80%), they may be reused for either the removal of the same or different contaminants [6], yet in the latter case, careful treatment/disposal is of the utmost importance to avoid secondary environmental contamination [7]. For example, their subsequent incineration to produce biochar can be a feasible handling alternative since the product obtained is capable not only of enhancing soil properties but also of serving as a fertilizer if previously used for nutrient removal [5,7]. Furthermore, post-adsorbents—such as biochar from the removal of heavy metals—may also work as supercapacitors or catalyst/catalyst supports, which could act as potential substitutes for expensive nanomaterials (e.g., carbon nanotubes) [5,6]. Therefore, discovering a secondary use for spent adsorbents would reduce the treatment costs, but it is important to ensure that adsorbed contaminants are not released [7]. Thus, in order to prevent the latter, spent adsorbents could be “encapsulated” as reinforcements for composite materials [8].

Over the past five years, more than four thousand studies have delved into the utilization of natural fibers as reinforcements in polymer composites, acknowledging their substantial impacts on mechanical properties. For example, polyvinyl alcohol reinforced with oil palm empty fruit bunch (OPEFB), representing a fraction of 3% by weight, demonstrated a notable 300% increase in stiffness compared to the base polymer [9]. Likewise, incorporating date palm leaf into thermoplastic matrices enabled the composite to attain mechanical properties akin to commercial wood regarding its hardness (52.65–57.05), impact resistance (1.56–1.95 kJ/m^2^), tensile strength (2.88–4.82 MPa), elongation (4.0–5.8%), and flexural modulus (1.68 to 1.91 GPa) [10]. Despite these advancements, challenges persist in natural fiber-reinforced composites, mainly due to issues related to matrix-fiber compatibility, often requiring chemical, physical, or biological treatment of the fibers. The attention garnered by biological treatments is due to their environmentally friendly characteristics [11]. These processes utilize particular microorganisms, such as bacteria and fungi, in conjunction with enzymes, to enhance the surface of the fiber. In certain scenarios, these improvements have proven to be more effective than those attained through chemical treatments. For example, the impact resistance of banana fiber composites increased by 113.6% and 120.6% when treated with a 20% concentration of laccase and xylanase, respectively, compared to the 98% increase observed with NaOH treatment [12].

Despite the heightened attention to investigating composites reinforced with natural fibers and their modifications, none have yet delved into the potential use of post-adsorbents. Considering that these spent materials from filter beds come into contact with microorganisms from wastewater, they could be classified as biologically treated reinforcements, and their integration could significantly enhance the overall properties of the composites [13]. In other words, in the course of filtration/biofiltration, microorganisms produce enzymes that eliminate undesirable substances such as waxes, lignin, and pectic compounds, among many other lignocellulosic elements from raw/modified agro-industrial residues [11,13]. As a result, their hydrophobicity is decreased, whereas their roughness is increased, which upgrades the interlocking reinforcement matrix and leads to the strengthening of the mechanical properties of the final composites [13]. Despite these benefits, biological treatments are not free from drawbacks, including long processing times and difficulty in obtaining specific microorganisms/enzymes on local markets [13]. For these reasons, the use of microorganisms from wastewater for simultaneous treatment (wastewater) and biological modification (reinforcement) could potentially overcome these issues.

**Figure 2:**
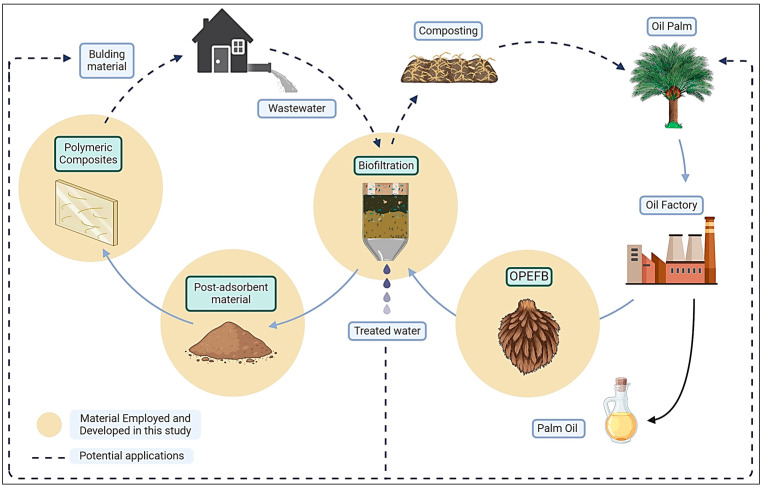
Application of OPEFBFs in the circular bioeconomy framework.

On the other hand, another pivotal point to assess is the role that the matrix plays in composite materials. Currently, studies in this field are evaluating the suitability of polymerizable thermoplastic resin matrices such as acrylic-based resins [14,15]. The origin of this trend lies in their remarkable features, such as their strong adhesion to different substrates, resistance to aging, light stability, effective pigment binding, easy application, and cost-effectiveness [16]. Considering the potential of acrylic resins and the utilization of agro-industrial residues in wastewater treatment, alongside the subsequent application of the resulting post-adsorbents within the circular bioeconomy (CBE) framework, this study aimed to assess whether saturated OPEFBFs could enhance the mechanical properties of the resultant compounds (Figure 2).

## 2. Materials and Methods

### 2.1. Materials

The oil palm empty fruit bunches were provided by the oil palm industry in Quinindé, Ecuador (0°20′ N, 79°29′ W). The matrix in the composites was SINTACRIL A-292^®^ liquid acrylic thermoplastic resin sourced from Poliacrilart, based in Quito, Ecuador. It has a density of 1.06 ± 0.01 g/cm³ and a Brookfield viscosity (SP1, 12 rpm) of 70 cP. 

### 2.2. Oil Palm Empty Fruit Bunches Conditioning

Oil palm empty fruit bunches were conditioned (cleaning, drying, grinding) and then sieved into three size ranges, 250–425 µm (mesh 40), 425–600 µm (mesh 30), and 600–850 µm (mesh 20), producing the R-OPEFBFs. The fiber lengths in the three size ranges follow a gamma distribution pattern, with a 69.72% overlap between them, notably more pronounced for meshes 20–30 (90.08%) and meshes 30–40 (79.07%) [17].

### 2.3. Utilization of Oil Palm Empty Fruit Bunches in Biofiltration Systems

P-OPEFBFs from biofilters (Øint = 8.1 cm, h = 100.0 cm) operated at two filter bed heights (90 cm, P1, and 60 cm, P2) for synthetic wastewater treatment, following the formulation by Almeida-Naranjo et al. [18]. The biofilters were inoculated with municipal wastewater from Quito (0°12′ S, 78°28′60″ W) before operation. The intermittently operated biofilters ran for 17 weeks (until material saturation), operating for 8 h/day for 5 days/week, with an average hydraulic head of 0.5 m^3^/m^2^d [19], treating wastewater with a COD of 589.71 ± 46.54 mg/L. The biofilters demonstrated removal efficiencies of 90.93% for COD, 88.37% for total nitrogen, and 77.2% for total phosphorus.

### 2.4. Fabrication of the Composites

**Figure 3:**
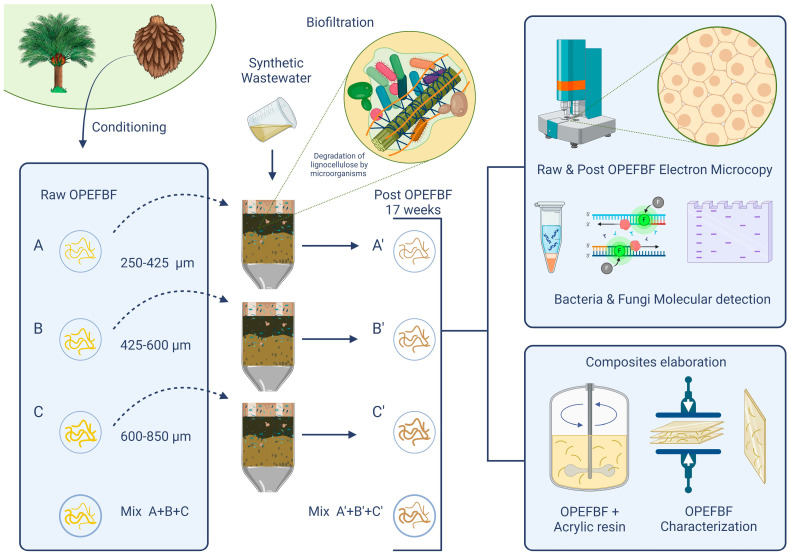
Methodology of research. A, B, C= R-OPEFBFs; A’, B’, C’ = P-OPEFBFs. A and A’ = 250–425 µm, B and B’ = 425–600 µm, C and C’ = 600–850 µm.

The R- and P-OPEFBFs, in three size ranges, were used, independently and mixed (33% of each size), as reinforcements. Mixing occurred at 400 rpm for 30 min at room temperature. After removing excess resin, the reinforcements were dried at 103 °C for 3 h. The embedded reinforcements were compression-molded at 100 °C and 150 bar for 40 min, yielding 150 *×* 150 *×* 2 mm composite sheets. The fiber-to-matrix ratio was 10:90 *w*/*w*% (Figure 3).

### 2.5. OPEFBF and Composites Characterization

The moisture content, extractives, lignocellulosic material, and ash content of the R-OPEFBFs were evaluated using ASTM standard methods. Specifically, the moisture content was determined according to ASTM D4442-20 [20], the extractives were analyzed following ASTM D1107-21 and ASTM D1110-21 [21,22], lignocellulosic material was measured using ASTM D1106-21 and ASTM D1109-21 [23,24], and ash content was assessed in accordance with ASTM E872-19 [25]. These standardized methods ensure accurate and reliable characterization of the R-OPEFBF samples.

#### 2.5.1. Instrumental Characterization of R/P-OPEFBFs

Morphological features of R/P-OPEFBFs were analyzed using scanning electron microscopy (SEM). The fibers were gold-sputter-coated using a QUORUM Q150R ES evaporator. A TESCAN electron microscope, model MIRA 3, at 5 kV, was employed. Semiquantitative elemental analysis was performed in the SEM chamber using a Bruker X-Flash 6|30 detector for Energy-Dispersive Spectroscopy (EDS) with a 123 eV resolution at Mn Kα.

The crystallinity of the R/P-OPEFBFs was assessed via X-ray diffraction (XRD). The XRD patterns were acquired with a Panalytical EMPYREAN model diffractometer, employing a copper X-ray tube (Kα, λ = 1.54056 Å) at 45 kV and 40 mA. Diffractogram analysis averaged four measurements from 5° to 90° (θ–2θ, Bragg–Brentano geometry) with HighScore Plus software version 4.9. 

The composition and functional groups of both the raw and post-adsorbent OPEFBs were determined using derivative thermogravimetric analysis (DTG) and Fourier transform infrared spectroscopy (FTIR), respectively. DTG analysis was performed using a METTLER TOLEDO TGA-2 thermal balance instrument over a temperature range of 25 to 750 °C, with a heating rate of 10 °C/min and a nitrogen flow of 50 mL/min. For FTIR analysis, a JASCO FT/IR-C800 spectrometer was utilized, performing twenty-five scans in the range of 4000 to 400 cm^−1^ with a resolution of 2 cm^−1^.

#### 2.5.2. Molecular Detection of Microorganisms in OPEFBF Composites

Microorganism detection in the R/P-OPEFBFs involved homogenizing the fibers in a TE buffer for 5 min, followed by efficient total DNA extraction using a thermal shock method. The buffer sample was homogenized, mixed with TE buffer, and frozen at −20 °C. Then, the cell suspension was heated to 95 °C for 5 min to denature the components and release the DNA. After brief centrifugation, the DNA-containing supernatant was stored at −20 °C for analysis [26].

The PCR for bacterial detection utilized the Eppendorf thermocycler Mastercycler^®^ Gradient (Eppendorf, Hamburg, Germany), following the specified conditions, and targeted the 16S rRNA fragment using the forward primer 8F (5′AGAGTTTGATCCTGGCTCAG3′) and the reverse primer 534R (5′ATTACCGCGGCTGCTGG3′) [27]. Fungi and yeast detection involved the use of the forward primer ITS1 (5′TCCGTAGGTGAACCTGCGG3′) and the reverse primer ITS4 (5′TCCTCCGCTTATTGATATGC3′). These primers targeted the internal transcribed spacer 1 (ITS1) and the 5.8S ribosomal RNA ITS2 [28]. PCR products were analyzed on a 2% agarose gel with SyBr Safe DNA stain (Invitrogen, Waltham, MA, USA) in 1X TBE buffer. Electrophoresis was run at 100 V for 35 min (Labnet Enduro Gel XL Electrophoresis System, Labnet International, Inc., Edison, NJ, USA). Gel images were captured under UV light with a ChemiDoc™ Imaging Systems gel documentation system (Bio-Rad, Hercules, CA, USA). The amplicon size was determined through comparison to a 100 bp DNA ladder (Invitrogen, Waltham, MA, USA).

#### 2.5.3. Composite Characterization

Tensile properties (elongation at break, tensile strength, elasticity modulus, and toughness) were evaluated per the ASTM D 638 standard [29]. An INSTRON universal testing machine, model 3365 (Norwood, Malvern, PA, USA), with a 500 N load cell and a crosshead speed of 20 mm/min performed the evaluations. The average values were determined by analyzing the results for ten Type IV dumbbell-shaped test specimens for each composite formulation.

## 3. Results and Discussion

### 3.1. Raw and Post-OPEFBF Characterization

The physicochemical analysis of the R-OPEFBFs reveals a lower water content (7.08 ± 0.11%) and extractive content (2.86 ± 0.21%), alongside elevated levels of cellulose (46.06 ± 1.06%), lignin (25.64 ± 2.49%), and hemicellulose (25.45 ± 1.14%). These lignocellulosic biopolymers make R-OPEFBFs candidates for applications as reinforcements in polymeric composites [8,30].

**Figure 4:**
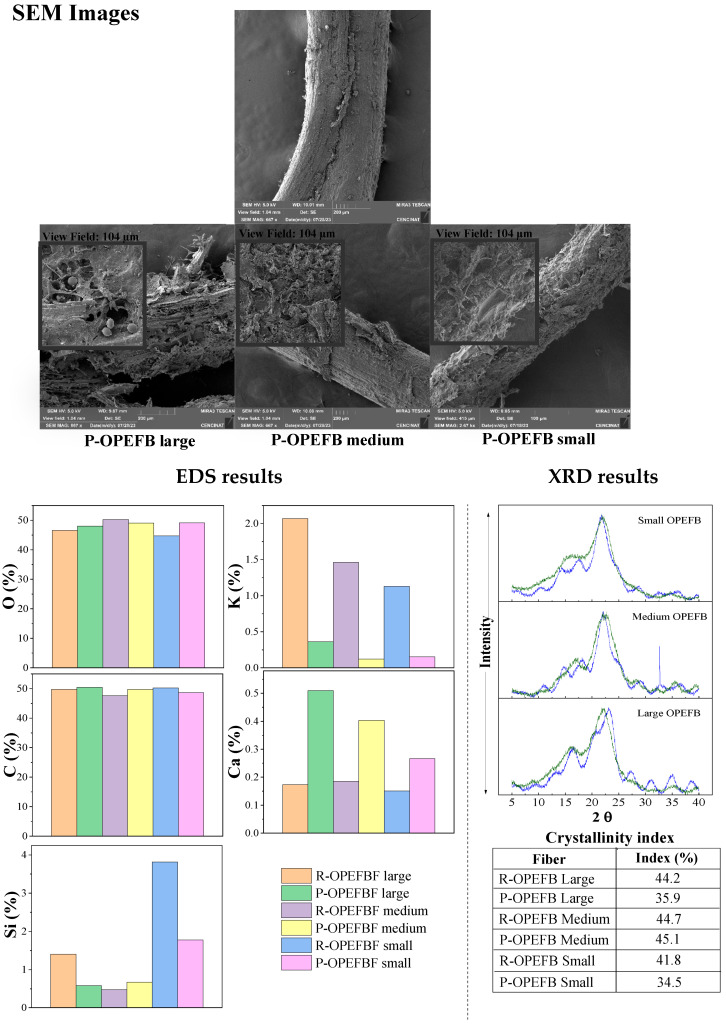
SEM, DRX, and EDS analyses of raw and post-OPEFBFs.

Figure 4 depicts SEM, EDS, and DRX analyses of the R- and P-OPEFBFs. The R-OPEFBF SEM images reveal intact fibers with a heterogeneous surface and a low pore count. Conversely, the P-OPEFBFs display broken fibers with rougher surfaces, more cavities, and striations. This fiber breakage confirms microbial-activity-induced molecular changes, such as cell wall breaking [31,32]. However, the porosity and fiber morphology changes are more visible in P-OPEFBF mesh 20 (large fibers). Yeasts can even be observed within the formed pores. In contrast, the morphological changes are less pronounced in P-OPEFBF meshes 40 and 60, and in fact, biofilm presence is observed around the fibers in OPEFBF mesh 60. In OPEFBF mesh 40, an average effect is observed. The increased presence of the pores could be linked to elevated loss of lignocellulosic material [33], probably caused by biodegradation [34].

**Figure 5:**
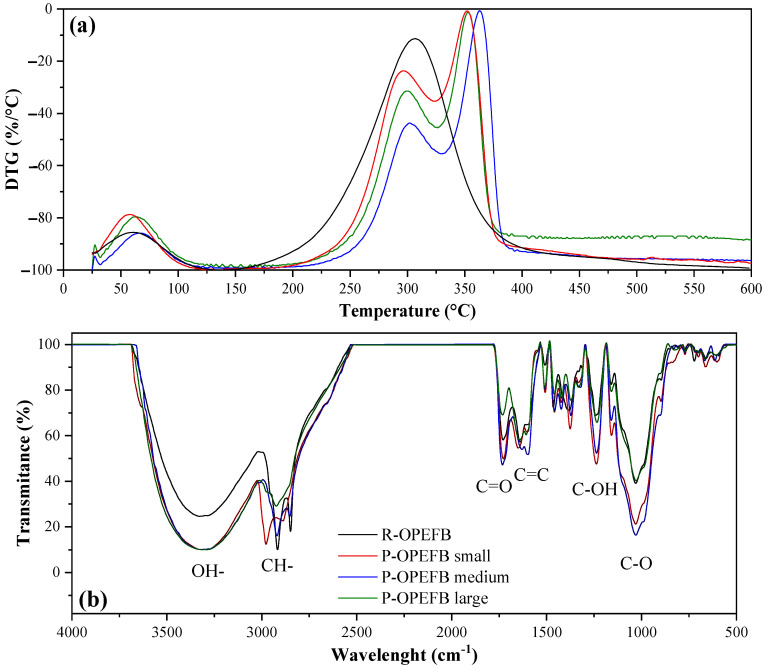
(**a**) DTG and (**b**) FTIR analyses of raw and post-OPEFBFs.

Figure 5 depicts the DTG (Figure 5a) and FTIR spectra (Figure 5b) of both the R- and P-OPEFBFs. The DTG analysis results reveal the first peak at temperatures below 150 °C in all the samples, attributed to water evaporation and the release of highly volatile compounds [35]. Between 150 and 410 °C, a peak is observed for the R-OPEFBFs, while two peaks are observed for the P-OPEFBFs, associated with the degradation of lignocellulosic material. The common peak, around 310 °C, is attributed to the removal of polyhydroxyl groups and the depolymerization/decomposition of lignocellulosic material [35,36]. The second peak in the P-OPEFBFs (around 350 °C) is associated with the presence of organic acids, aldehydes, ketones, partially degraded polysaccharides, phenolic compounds, and nitrogen-containing compounds. These compounds are byproducts of the microbial degradation of cellulose, hemicellulose, and lignin [37,38].

In the FTIR analysis, bands are observed around 3300 and 1650 cm^−1^ (O-H bond), between 2950 and 2890, and around 1370 cm^−1^ (C-H bond belonging to methyl groups), between 2920 and 2850 cm^−1^ (C-H bond belonging to methylene groups), and around 1700 cm^−1^ (carbonyl group bond) for the R-OPEFBFs. Additionally, characteristic bands of the glucosidic bond group around 1150 and 1030 cm^−1^ and of aromatic groups (Ar-R, C=C) between 1667 and 1450 and at 1244 and 667 cm^−1^ are present. These groups are associated with the presence of water, carboxylic acids, and lignocellulosic material, consistent with the physicochemical analysis. Both the amorphous (~800 cm^−1^) and crystalline (~1400 cm^−1^) phases of cellulose are observed [39]. Furthermore, changes in the intensity of several bands compared to the raw OPEFBFs are observed in the post-treated samples, associated with microbial activity during wastewater treatment in biofiltration systems. Yeasts have the ability to break down the fatty acid chains present in raw OPEFBFs through β-oxidation processes, thanks to the enzymes present in their peroxisomes [37]. Moreover, certain yeast species utilize cellulose and hemicellulose, particularly xylan, as carbon sources. During metabolism, cellulose is hydrolyzed into simple sugar molecules (glucose) due to the action of various enzymes, such as β-glucosidases, endo-β-1,4-glucanases, and exoglucanases [40]. The resulting glucose is converted into pyruvate through glycolysis, which, via various metabolic pathways, promotes the production of fatty acids. This is reflected in the increase in carbonyl group bands (around 1700 cm^−1^) [37,40].

Differences associated with the particle size of the P-OPEFBFs are observed in the FTIR spectrum. Larger particle sizes do not favor bacterial growth kinetics because the production of simple sugars is slower. Bacteria prefer smaller particles, as they facilitate the absorption and metabolism of nutrients due to their larger surface area. However, bacteria tend to consume less than they produce, leading to the accumulation of metabolic residues (biofilm shown in SEM images of Figure 4), composed mainly of sugars and proteins, causing observable changes in the FTIR spectrum [38].

Moreover, yeasts and other microorganisms can produce a wide range of metabolites through fermentation (e.g., the conversion of sugars into acids, alcohols, and gases) and the bioconversion of various organic compounds. The presence of microorganisms was confirmed through molecular analysis [37].

The molecular detection results for fungi (ITS) and bacteria (16S rRNA) revealed distinct bands—780 and 560 bands for fungi and bacteria, respectively—observed in the electrophoresis gels for P-OPEFBF meshes 40 and 60. Intriguingly, no bands were detected for the R-OPEFBFs of the same size. Conversely, no differences were observed between R-OPEFBF and P-OPEFBF mesh 20. In this context, it is important to emphasize that microorganisms have developed strategies for the degradation of recalcitrant components of lignocellulose such as lignin, hemicellulose, and cellulose, along with non-lignocellulosic materials such as waxes, pectin, proteins, carbohydrates, etc. [32,34,41]. Thus, effective microbial R-OPEFBF degradation could be performed by microbial enzymes, whole microorganisms, and microbial consortia. Notably, R-OPEFBF meshes 40 and 60 (lower size) are poised to exert a significant influence by serving as pivotal physical substrates that facilitate the establishment of microbial consortia, foster biofilm formation and consequently enhance the enzymatic degradation of lignocellulosic components within the vegetal material [34,42,43]. However, excessive particle reduction generates substances that slow down microbial activity (e.g., excessive volatile fatty acid production) and chemically alters the structure due to the small particle size [44].

These results are complemented by the XRD analysis (Figure 4), which reveals changes in fiber crystallinity. The R-OPEFBFs in the XRD analysis exhibit characteristic peaks of cellulose type II (identified by peaks at 2θ = 12, 16, 22.5°), common in lignocellulosic materials subjected to milling processes [32]. The peak around 16° corresponds to amorphous components like disordered cellulose, hemicellulose, and lignin, while the peaks at about 12° and 22.5° are associated with crystalline cellulose [45,46], which undergoes modifications following microbial activity. The crystallinity index of cellulose primarily showed changes in P-OPEFBF mesh 20 (around 8.3%) and P-OPEFBF mesh 60 (around 7.5%). P-OPEFBF mesh 40, on the other hand, displays slight increases in its crystallinity (0.4%). Previous studies have suggested that fungal activity can break down the crystalline portion of cellulose, facilitating cellulase access to β-glucosidic bonds during enzymatic hydrolysis [46,47]. Additionally, in the P-OPEFBFs, peaks beyond 22.5° indicate a loss of hydrogen bonds within and between molecules, potentially leading to the formation of a new crystalline structure [46].

**Figure 6:**
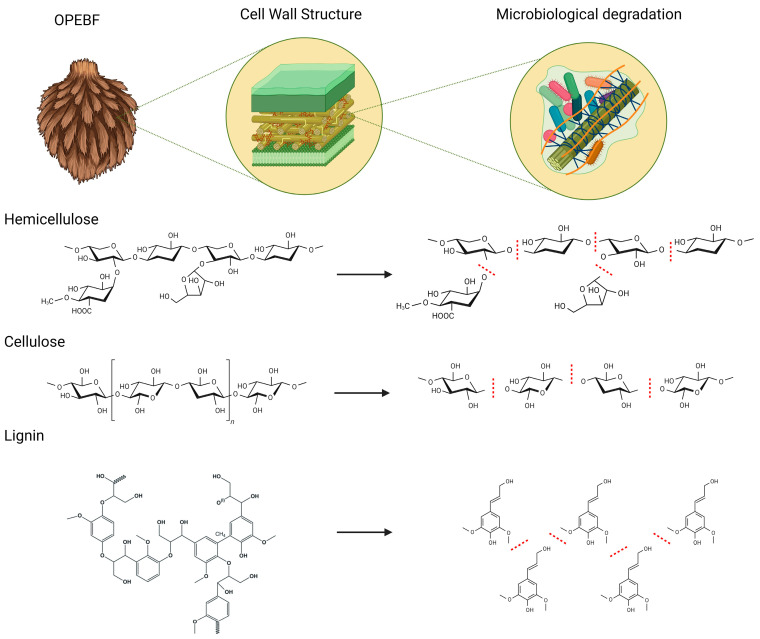
Potential breakage of cellulose, hemicellulose, and lignin chains in R-OPEFBFs due to microorganism action. Red dot lines: breakdown of molecules for microbial action [3,4].

The EDS analysis (Figure 4) shows that the carbon (C) and oxygen (O) concentrations remain nearly constant, despite biological modifications to the fibers. The stability in C and O levels can be attributed to the potential washing effect of water circulating through the biofilter, enhanced by microbial growth and biofilm formation. However, variations in silica content are evident in the R-OPEFBFs, with higher levels in the mesh 60 fibers due to the more extensive milling process required to achieve smaller fibers, exposing silica to a greater extent [32]. In contrast, the P-OPEFBFs exhibit reduced the silica content, linked to their interaction with lignin. Essentially, the loss of lignin results in a loss of silica molecules. Moreover, the decreased silica content may facilitate cellulose hydrolysis, as the silica structure acts as a physical barrier impeding cellulase penetration [45]. The increase in calcium in the P-OPEFBFs is associated with microorganisms interacting with wastewater and the fibers, resulting in mineral precipitates, primarily composed of calcium carbonate [48]. Conversely, the decline in potassium is linked to its utilization in microorganism metabolic processes, including osmotic pressure regulation and protein synthesis, leading to a reduction in its concentration in the fibers [49]. As a complement to the observed changes in the instrumental analysis of the R/P-OPEFBFs, Figure 6 illustrates the potential breakage of the main component (lignocellulosic material) chains in the R-OPEFBFs caused by degradation from microorganisms present in the wastewater.

### 3.2. Composite Characterization

**Figure 7:**
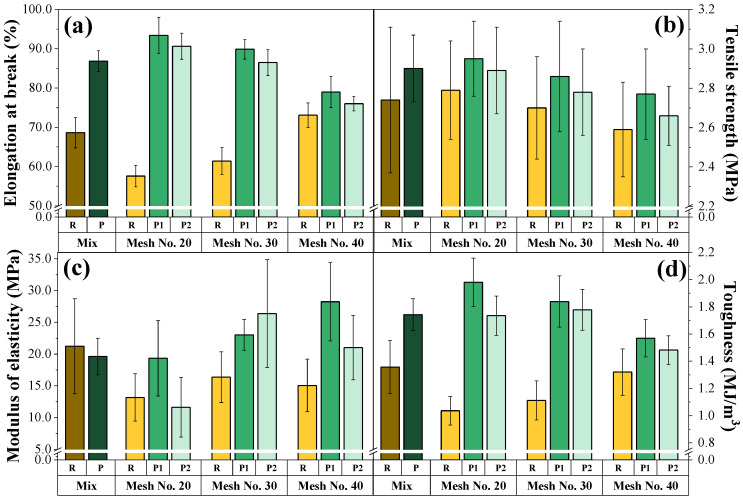
Mechanical properties of composites (**a**) Elongation at break, (**b**) Tensile strength, (**c**) Modulus of elasticity, (**d**) Toughness. Olive green: R mix, dark green: P mix, yellow: R in its different particle sizes, green: P1 in its different particle sizes, light green: P2 in its different particle sizes. R = raw OPEFBFs, P = post-OPEFBFs, P1 = post-OPEFBFs from 60 cm biofilter, P2 = post-OPEFBFs from 90 cm biofilter.

Figure 7 illustrates the mechanical characterization of the composites and reveals a correlation between the mechanical properties and the size of the OPEFBFs. Smaller sizes of R-OPEFBFs favor certain mechanical properties of the composite, which decrease as the fiber size increases. The smaller R-OPEFBFs (250–425 µm) exhibit superior elongation at breakage, showing significant differences (*p*-value ≤ 0.05), and toughness (1.3 MJ/m^3^, respectively), without significant differences. This disparity is attributed to the improved interaction/compatibility between the smaller fibers and the matrix [8]. However, they demonstrate a comparable tensile strength (2.6 MPa) to the other medium-sized (2.7 MPa) and large (2.8 MPa) fibers. Similarly, the modulus of elasticity of the small R-OPEFBFs (15.1 MPa) is comparable to that of the medium-sized particles (16.4 MPa), without significant differences. The behavior in terms of the tensile strength and the modulus of elasticity could be associated with the fiber distribution in all three sizes, as proven in our previous study [17].

On the other hand, the P-OPEFBF composites exhibit significantly enhanced mechanical properties relative to the R-OPEFBF composites, with elongation at break, tensile strength, modulus of elasticity, and toughness increasing by 3.9–36.5%, 2.6–3.5%, up to 37.9, and 10.9–40.3%, respectively. These improvements stem from the biological modification experienced by R-OPEFBFs during wastewater treatment. The rougher and more porous surface of the P-OPEFBFs (Figure 4) enhances the mechanical interlocking between the fibers and the matrix. This is attributed to the increased porosity of the fibers, resulting from the biodegradation of lignin and lignocellulose, which exposes more microfibrils [12,50]. Consequently, the matrix can penetrate and diffuse more effectively into both the pores and the spaces between the fibers [12]. Additionally, the breakdown of lignin, cellulose, and hemicellulose chains (Figure 5) creates a charged surface that facilitates the formation of covalent bonds between the fibers and the polymeric matrix. Another significant factor contributing to the mechanical properties is the composition of microorganisms, which promote the formation of adhesive bonds through reactions such as esterification during the thermal processing involved in composite fabrication [50].

These phenomena are reflected in an increase in the stress transfer capacity in the composites and therefore in an enhancement of their mechanical properties. However, P2 composites in all fiber sizes exhibit lower mechanical properties than P1, with no significant differences (*p*-value > 0.05). This effect is attributed to the deeper biofilter bed in P2, which increases the hydraulic retention time and the contact time between wastewater and the contaminated fibers, resulting in greater changes (non-controlled modification) in the R-OPEFBFs [51]. This degradation was evident in treated water, as the concentration of organic matter in the P2 biofilter-treated water was higher than that in the P1 biofilter-treated water. Furthermore, a mixture of R-OPEFBF sizes exhibits superior mechanical properties compared to 425–600 and 600–850 µm fibers. This likely occurs because different-sized fibers can intermingle, achieving a better reinforcement distribution.

## 4. Conclusions

The instrumental characterization (SEM, EDS, XRD, FTIR, and DTG) of the fibers reveals significant changes in the R-OPEFBFs induced by microbial activity from the microorganisms present in wastewater, particularly pronounced in the larger R-OPEFBFs (600–800 µm). These changes translate into a notable improvement of up to 40.3% in the mechanical properties of the composites reinforced with P-OPEFBFs. These findings underscore the technical feasibility of using biologically modified post-adsorbents derived from microorganisms present in wastewater, opening up promising prospects for their application in polymeric composites. This not only emphasizes the technical feasibility of utilizing post-adsorbents in the production of sustainable composite materials but also contributes to closing economic and environmental loops by integrating biological processes into material production. Therefore, in future studies, the potential application of the composites reinforced with P-OPEFBFs and their performance in operational environments will be evaluated. Additionally, we intend to analyze how the presence of other contaminants (such as heavy metals, dyes, and emerging contaminants) in the post-adsorbents could influence the mechanical properties of the composites obtained. Furthermore, efforts are aimed at optimizing the manufacturing process to enhance the mechanical properties and overall performance of the polymeric composites.

## Data Availability

The original contributions presented in the study are included in the article, further inquiries can be directed to the corresponding authors.
